# PATCH: posture and mobility training for care staff versus usual care in care homes: study protocol for a randomised controlled trial

**DOI:** 10.1186/s13063-018-2863-5

**Published:** 2018-09-24

**Authors:** Liz Graham, Robert Cicero, David Clarke, Bonnie Cundill, Alison Ellwood, Amanda Farrin, Jill Fisher, Madeline Goodwin, Rebecca Hawkins, Karen Hull, Claire Hulme, Dominic Trépel, Rachel Williams, Anne Forster

**Affiliations:** 1 Academic Unit of Elderly Care and Rehabilitation, Bradford Teaching Hospitals NHS Foundation Trust, Bradford, UK; 2 Clinical Trials Research Unit, University of Leeds, Leeds, UK; 3 Academic Unit of Elderly Care and Rehabilitation, Bradford Teaching Hospitals NHS Foundation Trust and University of Leeds, Bradford, UK; 4 Leeds Neurophysiotherapy, Rawdon, Leeds, UK; 5 Academic Unit of Health Economics, Leeds Institute of Health Sciences, University of Leeds, Leeds, UK; 6 Trinity College Dublin, Dublin, Ireland

**Keywords:** Care homes, Nursing homes, Older people, Feasibility, Cluster randomised controlled trial, Staff training, Physiotherapy, Posture, Mobility, Process evaluation

## Abstract

**Background:**

Residents of care homes have high levels of disability and poor mobility, but the promotion of health and wellbeing within care homes is poorly realised. Residents spend the majority of their time sedentary which leads to increased dependency and, coupled with poor postural management, can have many adverse outcomes including pressure sores, pain and reduced social interaction. The intervention being tested in this project (the Skilful Care Training Package) aims to increase the awareness and skills of care staff in relation to poor posture in the older, less mobile adult and highlight the benefits of activity, and how to skilfully assist activity, in this group to enable mobility and reduce falls risk. Feasibility work will be undertaken to inform the design of a definitive cluster randomised controlled trial.

**Methods:**

This is a cluster randomised controlled feasibility trial, aiming to recruit at least 12–15 residents at each of 10 care homes across Yorkshire. Care homes will be randomly allocated on a 1:1 basis to receive either the Skilful Care Training Package alongside usual care or to continue to provide usual care alone. Assessments will be undertaken by blinded researchers with participating residents at baseline (before care home randomisation) and at three and six months post randomisation. Data relating to changes in physical activity, mobility, posture, mood and quality of life will be collected. Data at the level of the home will also be collected and will include staff experience of care and changes in the numbers and types of adverse events residents experience (for example, hospital admissions, falls). Details of NHS service usage will be collected to inform the economic analysis. An embedded process evaluation will explore intervention delivery and its acceptability to staff and residents.

**Discussion:**

Participant uptake, engagement and retention are key feasibility outcomes. Exploration of barriers and facilitators to intervention delivery will inform intervention optimisation. Study results will inform progression to a definitive trial and add to the body of evidence for good practice in care home research.

**Trial registration:**

ISRCTN Registry, ISRCTN50080330. Registered on 27 March 2017.

**Electronic supplementary material:**

The online version of this article (10.1186/s13063-018-2863-5) contains supplementary material, which is available to authorized users.

## Background

Life expectancy has increased dramatically over the last century. As the population ages, the numbers at the oldest ages will increase the fastest. In mid-2016, there were 1.6 million people aged 85 years and over; by mid-2041, this is projected to double to 3.2 million [1]. One consequence is an increase in the demand for long-term care which, despite the increased emphasis on community care [2], will remain a necessary component of health and social care provision [3]. The need for care home places is predicted to rise by 150% in the next 50 years [4]. Increasing age is associated with increasing disability. A United Kingdom (UK) survey [5] reported that 89% of residents of care homes required care because of disability from long-term conditions, 72% had mobility problems and 62% were confused.

Residents of care homes are among the frailest in our population with significant health and social care needs [6]. The health requirements of residents place considerable burden on the UK National Health Service (NHS) and greater demands are placed on the workload of general practitioners (GPs) providing care for care home residents than caring for people in their own homes [7]. Care home residents are significantly more likely to attend emergency departments by ambulance and be admitted to hospital compared to the older population generally [8]. Hospital admission exposes residents to the risk of nosocomial infections and falls, and is disruptive for this frail population as they struggle to return to their previous health state once discharged.

The promotion of the health of frail older people in care homes is poorly and inconsistently developed. Provision of programmes which could promote health and wellbeing within UK nursing homes is only patchily realised [9]. A 2001 survey showed that only 10% of care home residents receive physiotherapy and just 3% occupational therapy [10]. It is reported that care home residents spend the majority of their time inactive [11–13] with low levels of interaction with staff. Previous work by members of this team [14] indicated that residents spent up to 13 h of their waking day sedentary. Sedentary behaviour is adversely associated with chronic disease risk factors and all-cause mortality [11]. Decreasing mobility, increasing dependency and poor postural management have many adverse effects. For residents in care homes, this may lead to increased incidence of pressure sores, pain, contractures and loss of independence [15], reducing opportunities to participate in social activities. Social isolation negatively impacts on mood and self-esteem, which can then further adversely affect physical health [16].

A Cochrane Review of rehabilitation in long-term care [17] reported that it was possible to improve physical activity in this population but that interventions were often time-limited and resource-intensive. A potentially more sustainable approach is to enhance the culture in care homes. To address this, a group of senior physiotherapists developed and piloted a manualised, competency-based training programme focused on posture and mobility called the Skilful Care Training Package (SCTP), which showed initial promise in raising awareness and providing practical skills training to staff in early pilot work. This paper describes the protocol for the feasibility trial evaluation of the SCTP compared with usual care (UC), within care homes.

## Methods / design

### Design summary

**Fig. 1:**
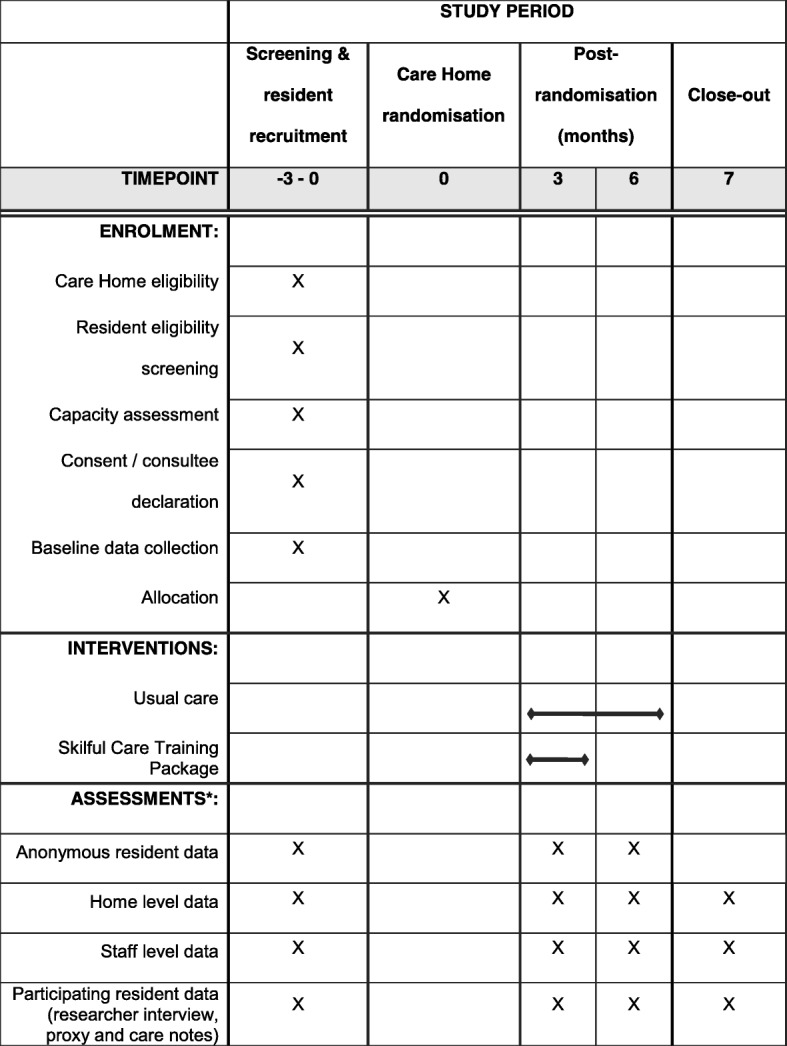
SPIRIT figure. *Further detail is provided in Table 1

The PATCH trial is a multicentre, two-arm, pragmatic, cluster randomised controlled feasibility trial. A summary of the trial design is illustrated in Fig. 1.

### Aims and objectives

**Fig. 2:**
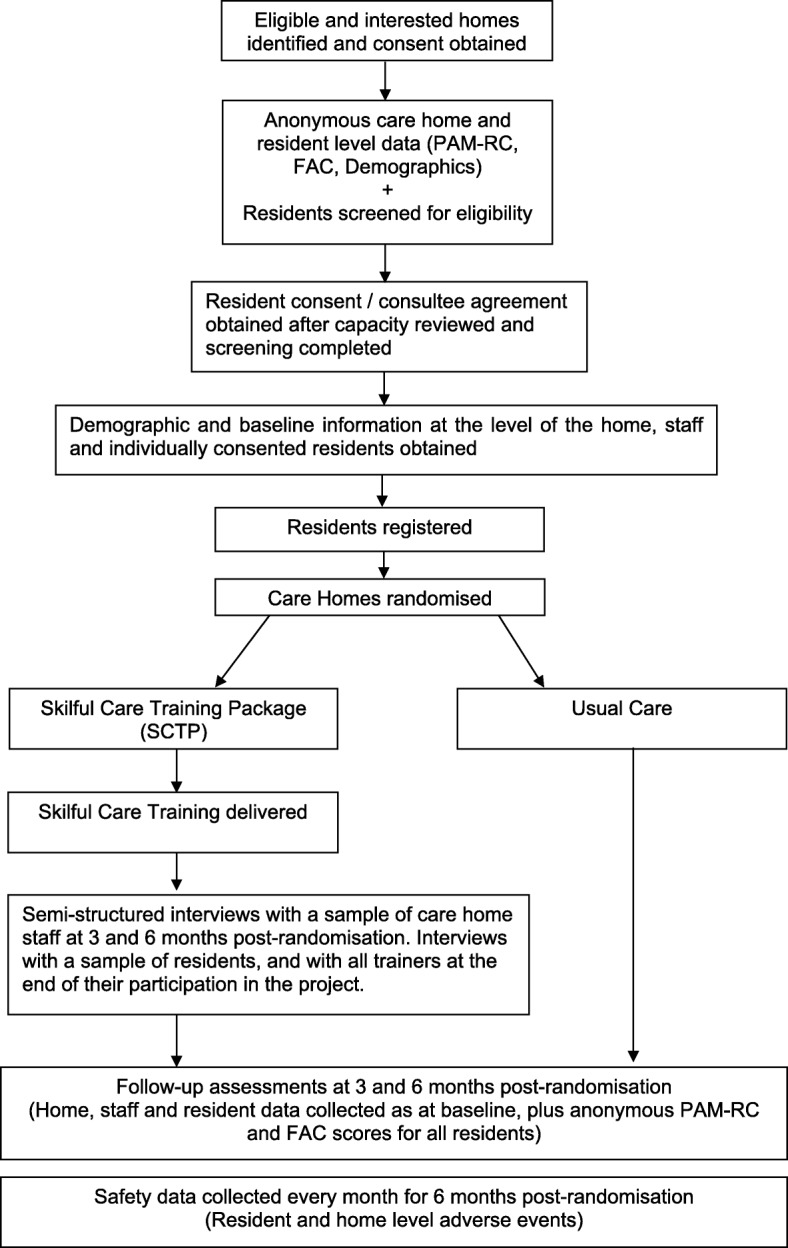
Trial flow diagram

The aim of this trial is to assess the feasibility of conducting, and to inform the design of, a definitive randomised controlled trial (RCT) to investigate the clinical and cost-effectiveness of the SCTP compared with UC, within care homes. Figure 2 illustrates trial processes from screening to follow-up.

The objectives are to:

1. Ascertain recruitment and retention rates at the level of the care home, staff and residents.
2. Assess feasibility and acceptability of follow-up (retention of participants and completeness of data).
3. Assess the appropriateness of outcome measures to inform the choice of primary and secondary outcomes for the main trial.
4. Explore and clarify procedures to collect anonymised data at the level of the home.
5. Assess the feasibility and best methods of collecting resource and outcome data for a future large-scale cost-effectiveness analysis. This will also involve exploration of the reliability of routinely collected NHS and care home data.
6. Monitor relevant adverse events (AEs) and confirm how best to collect these.
7. Assess the feasibility of delivering the SCTP.
8. Develop methods to assess compliance with and adherence to the SCTP.
9. Gain understanding of the barriers and enablers to delivering and implementing the SCTP in care homes to optimise implementation in the main trial.
10. Obtain understanding of care home staff and trainers’ views of the SCTP to inform refinement for the main trial.
11. Obtain insight from residents as to the acceptability of the SCTP to inform refinement for the main trial.
12. Assess evidence of proof of concept relating to potential efficacy.
13. Ascertain the number of care homes and residents needed for a definitive trial.
14. Estimate the intra-class correlation coefficient (ICC) for the selected primary outcome from the trial data and from other literature and data sources.

### Recruitment setting

Ten residential and/or nursing care homes with residents aged 65 years or older in the counties of West and North Yorkshire in the UK will participate. These locations were selected to fit logistically with intervention delivery and research data collection. This geographical area covers a range of multi-ethnic rural and urban populations, so ensuring generalisability of findings. All care homes within these counties will be identified from the Care Quality Commission (CQC) care directory (accessed via www.cqc.org.uk). They will be approached if they have no more than four improvements on their latest CQC inspection report and no inadequate ratings. First contact with homes will involve sending a study flyer and summary information sheet to care home managers and following this up with a telephone call where no response is received. In addition to this ‘mail shot’ approach, the trial will be publicised at suitable local forums for care home managers and organisations and also by word of mouth by the trial team when they have opportunistic contact with care homes. An initial eligibility check will be undertaken over the telephone with managers at interested homes. If this check is positive, it will be followed by a visit to establish full eligibility and discuss trial procedures.

These recruitment processes will continue until 10 homes have been identified.

### Care home eligibility

Care homes (as defined above) will be eligible to take part if they meet all the following inclusion criteria:

1. The manager has been in post for at least six months and is likely to remain in post for the study period.
2. The manager is willing and able to release staff to attend SCTP training sessions and to contribute to data collection.
3. Training in manual handling is provided to all direct care staff (a prerequisite for the SCTP training).
4. It is anticipated that 12–15 eligible residents could be recruited to take part in the trial.

Care homes are not eligible to take part if they meet any of the following exclusion criteria:

1. Is unsuitable for inclusion due to being subject to CQC improvements in areas relevant to the trial, enforcement notices / inadequate ratings, admissions ban, or relevant moderate or major compliance breaches.
2. Is receiving special support for specific quality concerns (e.g. serious safeguarding investigations, voluntary or compulsory admissions bans).
3. Physiotherapy training which involved postural management and physical activity has been delivered to staff in the last 12 months.
4. The SCTP has already been delivered to the home.
5. Is taking part in another trial or initiative which would conflict with the SCTP and / or with data collection required for this trial.

In larger homes, a particular unit (e.g. nursing unit) and the associated pool of staff may take part, rather than the whole home.

### Resident eligibility

Following care home agreement to participate, all residents will be screened for eligibility (anonymously to the trial team). Researchers will discuss resident eligibility with care home staff who will identify those who meet the inclusion criteria and record reasons for ineligibility.

All residents within the home (or participating unit) will be exposed to the intervention, should the home be allocated the SCTP; thus all may potentially benefit from enhanced staff knowledge and skills. Residents who are more likely to benefit from staff skills acquired during SCTP training, and where there are no confounding factors present that may affect outcomes, will be identified. Thus residents who are aged 65 years or older and are permanent residents within the home will be considered for participation in the data collection element of the trial, whereas those who are independently mobile (a Functional Ambulatory Classification [FAC] [18] score of 5 or 6), or who are currently having a course of physiotherapy, occupational therapy or speech and language therapy, or who have a life expectancy of less than three months will not be eligible to take part. It is anticipated that, should the intervention be effective, it would benefit residents regardless of mental capacity. Excluding those lacking capacity would affect the generalisability of the trial results and thus all eligible residents (with and without capacity) will be approached in line with the provisions of the Mental Capacity Act (MCA) [19].

### Resident recruitment and consent

The home manager or senior staff member will undertake an initial assessment of the capacity of each eligible resident to consent to take part in the trial. All residents will be assumed to have capacity to consent unless assessed to lack capacity in accordance with MCA guidance.

Where residents have capacity, care home staff will introduce the trial concept to them. If they are willing to consider involvement and agree to speak to the researcher, the researcher will discuss the trial in more detail and provide information about the study. Residents will be offered at least 24 h to consider involvement and consult with relatives, friends or staff if they wish. Thereafter, if they are still interested in participating, the researcher will obtain their written consent.

Where residents lack capacity, consultees will be identified and approached in line with procedures outlined in the MCA. Care home staff will identify a relative or friend who knows each resident well to approach to act as a personal consultee. The care home manager will send a study letter and information sheets to identified personal consultees, who will be given 10 days to respond before being sent a reminder letter and a further seven days to reply. They will be offered the opportunity to discuss the study with the researcher. Where a personal consultee does not respond or is unwilling to take on the role, a nominated consultee within the care home will be identified to provide their opinion on the resident’s wishes regarding study participation. Nominated consultees should be independent of the research, but in a care home setting this is difficult. As agreed with the Research Ethics Committee (REC), nominated consultees must not provide data about residents to maintain a level of independence from research procedures and should not, wherever possible, be directly involved in the resident’s care.

Capacity will be monitored on an ongoing basis, as will the availability of personal consultees. Any loss of capacity or changes in consultee would result in seeking a new consultee to facilitate continued participation.

### Staff involvement

Active involvement of care home staff is crucial to the trial. All nursing and care staff will be invited to take part in the study at each time point, with the exception of bank or agency staff who have worked in the home for less than one month during the preceding six months.

All eligible staff will be asked to provide data about themselves and their role, with some also asked to provide data about participating residents. If the care home receives the intervention, staff will be asked to attend training and will be invited to participate in the process evaluation interviews.

Staff members will be given a study information sheet before baseline data collection and again before each follow-up time point to ensure all currently employed care and nursing staff are aware of trial processes. Staff involved in specific roles (e.g. providing data about a resident) will be provided with a short supplementary information sheet about their role at the time of taking it on.

### Care home randomisation

Following confirmation of eligibility and consent (or consultee agreement), and subsequent to baseline assessments with/about participating residents, at the level of the home and from individual staff members, care homes will be randomised on a 1:1 basis to receive SCTP or UC. Randomisation will be performed by the statistical team at the Clinical Trials Research Unit (CTRU), using block randomisation with randomly selected block sizes. Concealment of sequencing is ensured by the physically separate locations of the CTRU and the blinded study researchers.

Following randomisation, the CTRU statistical team will inform the unblinded members of the research team of the randomisation outcome, so that arrangements for training in the intervention can be made for those homes allocated SCTP. Unblinded members of the research team will be informed of the random allocation of the last two homes at the same time, to avoid predictability of care home allocation and selection bias in the recruitment of homes and/or residents. An unblinded member of the research team will inform the care home manager of the treatment allocation by telephone and email. This same member of the team will arrange follow-up visits with managers in an effort to keep researchers undertaking outcome assessment blind to allocation throughout the trial. This will ensure researcher follow-up visits are not arranged at the same time as intervention training sessions within the home and will allow reinforcement of the need to not divulge allocation details.

To ensure sufficient outcome data for evaluation at six months, despite initial assessment that there should be 12–15 eligible residents at a participating care home, a minimum of eight participating residents will be accepted for homes to proceed to randomisation.

### Intervention

The SCTP is a competency-based, manualised training programme which evolved through review of the evidence, needs analysis and expressed requirements of care home staff.

The training course aims to increase the skills of care assistants and nursing staff in handling techniques (to facilitate movement) and to promote good positioning (to maintain functional posture) thus protecting body shape, avoiding choking, reducing pain and encouraging activity, so enabling residents to be as active and independent as they wish to be. The course is designed to be delivered to all those providing care (care assistants and nursing staff) rather than to be cascaded down to staff via the training of key workers. The course is practical in nature and emphasis is given to person centred care and the development of empathy towards residents.

SCTP will be delivered by expert physiotherapists (trainers) to staff in each of the five care homes randomised to the intervention arm. Two trainers are co-applicants who have developed the SCTP, and all four trainers are qualified physiotherapists who each have recognised training qualifications. Together they have optimised the training package for use within the trial and have undertaken detailed training and development work to ensure quality and consistency of delivery.

Course content is manualised to ensure consistency, but the emphasis will be adapted according to the particular needs of a home and individual staff members. This will be done in consultation with the care home management team after randomisation and before the first training session. The SCTP is generally presented in three 2.5-h sessions with a ratio of one trainer to a maximum of eight staff, but can be presented over two or one longer session(s) if this fits better with staff availability and training plans.

### Usual care

Control homes will continue to provide care as usual with no additional interventional input as a result of trial participation. No restrictions will be placed on homes undertaking additional training or development as part of their usual care. Data will be collected by researchers from care home managers at all 10 homes to describe usual care, any changes in practice over the duration of the trial, new procedures or care processes adopted by a home during the study period and staff turnover levels.

### Data collection

As the intervention will be delivered at the whole home level, it is important to assess the impact on the whole home. Thus data will be collected at the home level and from all care and nursing staff, as well as from and about participating residents. Data will be collected at baseline (before randomisation) and at three and six months post randomisation by blinded trial researchers who will visit each home at each time point. Maintenance of blinding will be monitored and reported by researchers as soon as they become aware of a care home’s allocation.

Personal details of participating residents will be held centrally, in accordance with the terms of consent or consultee agreement, to facilitate contact at follow-up time points. They will be held separately (both paper and electronic copies) to all outcome data.

A summary of assessments and outcome measures can be found in Table 1.

**Table 1:** Summary and timing of assessments

Assessment	Method of completion	Timeline			
		Screening	Baseline	3 months	6 months
Screening, eligibility and consent					
Care home screening and eligibility	Researcher assessment / Researcher Interview (M)	X			
Resident screening and eligibility	Staff assessment with researcher support (S)	X			
Anonymous resident data: Physical Activity and Mobility in Residential Care Scale (PAM-RC) and Functional Ambulatory Classification (FAC)	Staff assessment with researcher support (S)	X		X	X
Trial consent (including process evaluation) (Resident (incl. personal/nominated consultee))	Self-completion (R) or Consultee (S, RF)		X		
Contact details (Resident (incl. personal/nominated consultee), staff informant	Researcher assessment (R, S, SI, RF)		X	(X)	(X)
Process evaluation consent	Self-completion (S)			X	X
Care home level data					
Care home demographics (including staff profile and training)	Researcher interview (M)		X	X	X
Home level resident profile	Researcher interview (M)		X	X	X
Significant events (D&V, temporary closure, CQC findings, change of manager/owner) and staff welfare	Researcher interview with manager / delegate (M)			X	X
Adverse events: total no. resident falls, hospitalisations and deaths	Researcher telephone interview with manager / delegate (M)		Collected monthly		
Staff measures (about the member of staff)					
Demographics	Self-completion (S)		X	X	X
Posture and movement questionnaire	Self-completion (S)		X	X	X
Kiersma-Chen Empathy Scale	Self-completion (S)		X	X	X
Person-Centred Care Assessment Tool (P-CAT)	Self-completion (S)		X	X	X
Resident measures (about the resident)					
Self-reported pain (Iowa Pain Thermometer)	Researcher interview / self-completion (R)		X	X	X
Perceived health (EQ-5D-5 L)	Researcher interview (R)		X	X	X
Six-item cognitive impairment text (6-CIT)	Researcher interview (R)		X	X	X
Posture observation tool	Researcher observation (R)		X	X	X
Staff-completed measures (about the resident)					
Activities of daily living (ADL) Barthel Index	Researcher interview (SI)		X	X	X
Perceived health (EQ-5D-5 L Proxy)	Researcher interview (SI)		X	X	X
Continuing Care Ability Measure (CCAM)	Researcher interview (SI)		X	X	X
PAM-RC	Researcher Interview (SI)		X	X	X
FAC	Researcher Interview (SI)		X	X	X
Resident information from care home records / staff					
Co-morbidities	Researcher collection from notes		X	X	X
Healthcare resource use	Researcher collection from notes		X	X	X
New therapies (individual) started since baseline	Researcher collection from notes			X	X
Pressure sores, falls, hospital admissions	Researcher collection from notes		X	X	X
Usual care and intervention data					
Observations of staff and resident interactions	Researcher observation		X	X	X
Usual care details (inc. new procedures or care processes)	Researcher interview (M)		X	X	X
Intervention fidelity and adherence (training, content, delivery, attendance)	Physiotherapist assessment (S)			X	X
Process evaluation data					
Views of intervention (inc. enablers and barriers)	Researcher interviews (S, R)			X	X

*M* care home manager, *R* resident, *S* care home staff, *SI* staff informant, *RF* relative/friend

#### Care home level data

At each time point, the care home manager or a nominated staff member will be asked to provide data to inform the staff and resident profile of the home. This will include: ownership (independent or part of a chain); number of beds in the home; full-time equivalent number of staff; engagement with local services (for example, community matron); and presence of specialist units (for example, dementia units). They will also be asked to provide details of their manual handling policy, staff training and any new initiatives they may be undertaking.

Anonymous home-level data will be collected on mortality, residents’ pressure areas, number and duration of hospital admissions, number of visits by health professionals, and falls for the six months before randomisation and for the duration of a home’s participation. For safety monitoring purposes whole-home falls, hospitalisations and mortality will also be collected via a researcher telephone call to the care home manager on a monthly basis post randomisation.

At baseline, as part of the screening process, and again at each follow-up time point, anonymous PAM-RC [20] and FAC [18] data will be collected for all residents to establish the activity and mobility profile of the home, and to establish the representativeness of the resident population taking part in the trial.

#### Resident level data

Residents will complete the EuroQol 5-Dimension 5-Level (EQ-5D-5 L) [21, 22], the six-item Cognitive Impairment Test (6CIT) [23] and provide a self-reported pain assessment [24] via researcher interview, where they are able.

A purposely developed postural assessment tool will be completed via researcher observation of participating residents’ posture. This tool has been developed specifically for use in this trial after an extensive search revealed no appropriate existing tools to measure observed seated posture that would be suitable for use by non-expert staff (researchers who would not be able to physically examine the residents when observing them). Observation of the position of specific areas of the body (e.g. pelvis, thoracic spine) will occur at two time points separated by at least 1 h to allow assessment of any postural change or observation of sustained static seated position. Researchers will be trained in use of the tool by qualified physiotherapists.

The Activities of Daily Living (ADL) Barthel Index [25], Continuing Care Ability Measure (CCAM) [26] and EQ-5D-5 L proxy will be completed via researcher interview with each participating resident’s identified staff informant (a member of staff who knows the resident well).

Researchers will also collect information from care home notes for each recruited resident. This includes relevant co-morbidities, healthcare resource use, pressure sores, falls, NHS and social care contacts. In addition to collecting this data from care home notes, and in order to optimise data collection methods for the definitive trial, alternative data sources such as NHS Acute Trust records and NHS primary care notes systems will be explored. Participant information provision and consent includes these alternative methods of data collection. This will contribute to both the health economic analysis and safety profiles.

#### Staff data

All eligible care and nursing staff will be asked to complete a staff booklet which includes demographic data (e.g. role in the home, training undertaken), a purposely designed questionnaire to elicit their knowledge of posture and movement, the Kiersma-Chen Empathy Scale [27] and the Person-Centred Care Assessment Tool (P-CAT) [28]. These questionnaires have been chosen to reflect anticipated changes in knowledge, skills and attitude following SCTP training.

Staff will be asked to return completed questionnaire booklets in a sealed envelope, either directly by post to the research office or to a secure box located within the care home. Consent will be assumed by completion and return of the booklet.

#### Process evaluation

A mixed method approach will be adopted to explore the delivery and implementation of the SCTP.

Data regarding attendance, engagement, content and delivery of the SCTP will be provided by the trainers after every session in each of the five SCTP homes. This will be recorded on trial proformas which will allow reporting of adherence to the training content and schedule, as well as providing a summary of the level of staff understanding and engagement from the trainers’ perspective. Trainers will also have the opportunity to document any successes or difficulties, as well as deviations from the training plan, to allow for real-time reflection.

Observations of the delivery of a sample of the training will be conducted in each intervention home. Verbal consent for the researcher to observe the training will be obtained from trainers and care staff at the beginning of each session. If consent is not given by all members, the session will not be observed. The researcher will take brief summary notes during the training session and will develop these into expanded fieldwork notes as soon as possible after the session; this approach provides the opportunity to record data in more depth and detail than is possible during an observation [29]. Semi-structured interviews will be conducted with a maximum variation purposive sample [30] of participating care staff in the five intervention homes following the three- and six-month assessment points. The aim of this sampling approach is to explore how the intervention is perceived, understood and engaged with by different care home staff in the intervention sites at the different time points. The sample will vary in relation to staff roles (i.e. managers, qualified nurses, senior care staff and care assistants) and experience (i.e. length of time working in care homes). Interviews are anticipated to take approximately 45–60 min and will be conducted until the point of data saturation [31]. Interviews will cover topics such as: views of the training; what worked (success factors); what was less successful; knowledge; perspectives and attitudes towards residents’ posture and staff’s handling skills; and reflections on perceived or actual change in practice. After the final training session has been delivered, the trainers will also be asked to participate in a semi-structured interview that will explore their views and experiences of the training, elements that worked well and not so well, and their views on how care staff received the training. In order to obtain insight into residents’ views of their daily routines in the care homes and their experiences of any changes following the introduction of the intervention, informal conversational qualitative interviews will be undertaken with a purposive sample of residents who have participated in the trial and who have given informed consent as part of their agreement to trial participation. A sample of 8–12 residents in total, with differing physical and cognitive impairments drawn from the care homes participating in the process evaluation, will be sought. The interview format will be flexible in nature (in relation to the length of time the interview takes and the manner in which the researcher engages with the residents) in order to engage residents who may have differing communication and cognitive abilities. Interview topic guides will be devised drawing on the published literature relating to care home care practices and skills training and in consultation with the research team. Topic guides may be refined in light of ongoing data collection and analysis. Interviews will be conducted in a quiet private area and, with permission, audio-recorded. Informed consent specific to interview participation will be obtained from care staff and trainers.

To allow reporting of intervention enactment (using new skills in practice) in a future definitive trial, a trial-specific observational tool designed to record instances of care behaviours reflective of skills (that could have been) learned during SCTP training will be developed and tested in more than one site. A process consent approach will be used for these observations, with researchers explaining their purpose to all individuals present on each occasion observations take place. Verbal consent from staff, care home residents and visitors will be sought on each occasion observations are undertaken. Observations will only take place in public areas of the care homes and only if there is no objection from anyone within that area.

#### Economic evaluation

The feasibility work will be used to test the performance of the data collection forms and methods. For the EQ-5D-5 L (and proxy EQ-5D-5 L) it is anticipated that performance will include assessment of clarity of concept (as evidenced by number of missing data (> 75% completed)). Integral to this part of the study is the acceptability of the EQ-5D-5 L (and proxy EQ-5D-5 L) to residents and their staff informants, evidenced by completion.

The feasibility of obtaining routinely collected health service use data from hospital records and GP administrative systems (e.g. SystmOne) in the timeframes required will be assessed. This will be compared against collection of data from care home notes (as described earlier) to assess the optimal methods of data collection for this population and in this setting.

Training details (number of sessions, number of attendees, number of trainers and duration of sessions) will be collected in order to provide a detailed breakdown of the cost of delivering the intervention.

### Sample size

The trial is designed to determine feasibility, not to evaluate effectiveness; therefore a formal power calculation is not appropriate. However, since we wish to assess whether change in our proposed primary outcome for a definitive trial, the PAM-RC, is feasible it is important that we have sufficient participants to make a preliminary and non-definitive randomised comparison of SCTP with UC. Our target sample size of 10 homes, each with 12–15 residents, allows us to detect a minimally important clinical difference of 0.5 standard deviation (SD) units with 80% power, a more relaxed false-positive error rate of 0.20 appropriate at this stage of the evaluation, 25% loss to follow-up and an ICC of 0.03 to 0.05.

### Analysis

#### Quantitative analysis

The analysis plan outlined here will be reviewed and a final, more detailed statistical analysis plan will be written before any analysis is undertaken. Any changes to the finalised analysis plan and reasons for change will be documented. Final analysis will be carried out when all available outcome data have been received. Analyses will focus on descriptive statistics and confidence interval (CI) estimation, with the exception of potential efficacy.

The recruitment strategy will be assessed by summarising the screening, eligibility, consent and randomisation stages at both the care home and resident level. Care home and staff demographics will be summarised to inform the context in which the trial was conducted.

Delivery of the SCTP will be evaluated by summarising staff attendance at the sessions, as well as feedback on engagement, content and delivery of the sessions. This will be assessed alongside the qualitative analysis from the process evaluation.

To assess feasibility and acceptability of follow-up, retention of homes and residents—including the number, timing and reasons for withdrawal—will be reported by trial arm to examine whether there are any systematic differences between the arms which could be attributed to the intervention. The difference in follow-up rates between the arms and corresponding CI will also be reported.

To inform the choice of primary and secondary outcomes for a definitive trial, completeness of outcome data will be summarised overall and by arm to assess both the acceptability of the measures and methods for collecting the data in a definitive trial. Point estimates and variability (SD) of the outcomes at each time point will be calculated by arm, together with 80% CIs for the difference in outcomes between the arms at six months using methods appropriate for cluster randomised trials with a small number of clusters [32]. The amount of missing data will also be presented.

To obtain a preliminary and non-definitive randomised comparison of the SCTP with UC for the PAM-RC, hypothesis testing will be conducted at the 20% significance level using the t-test on care home (cluster) level summaries. Adjustment for covariates will be carried out using a two-stage process. At the first stage, a standard regression model including the covariates of interest, but excluding the intervention effect, will be fitted to calculate cluster-specific expected values. Expected and observed values will be compared by computing a residual for each cluster. These cluster level residuals will then be analysed using methods based on the t-test in the second stage of analysis [31].

To inform the sample size/power calculation for a definitive trial, the SD of the PAM-RC score in the intervention and control arms will be assessed to confirm the SD to be used. The minimum clinically important difference will be established via the mean scores (and 80% CIs) in the control arm and by seeking clinical opinion. An estimate of the clustering effect (ICC) and 95% CI as well as cluster-size variation (coefficient of variation) relating to the PAM-RC will also be provided.

AEs, pressure sores, number of resident falls, hospitalisations and deaths will be summarised by arm to assess the safety of conducting a trial in this setting. Staff physical health issues and sickness levels will also be summarised by arm to ensure that the intervention is not having a negative impact on staff wellbeing.

#### Qualitative analysis

Transcribed interviews and fieldwork notes will be entered into NVivo computer software to facilitate management of a large dataset and the analytic process. A thematic framework approach [33] to data analysis will be employed to develop understanding of the facilitators and barriers to implementation of the SCTP in the care homes. Particular attention will be paid to staff’s views and experiences of the programme as well as the factors that facilitate or inhibit delivery or sustainability of the intervention over time. Findings will inform the development of the implementation process for a full-scale trial.

#### Economic analysis

While the primary aim of the economic analysis is to test the feasibility of data collection for any subsequent RCT, analysis of the data collected will include descriptive statistics of the resources used. Unit costs for health service resources will be obtained from national sources such as the PSSRU [34], the BNF [35] and NHS Reference cost database [36]. The perspective of the NHS will be adopted.

#### Progression criteria for continuation to a definitive randomised controlled trial

Guidelines for progression to a definitive RCT are based on a traffic light system of green (proceed to RCT design), amber (review RCT design and/or implementation, then proceed) and red (stop and do not proceed). Progression would be contingent upon meeting success criteria in the areas of participant recruitment, intervention delivery, data collection and follow-up. Progression criteria are detailed in Additional file 1.

### Trial organisation and governance

The PATCH trial is sponsored by the Bradford Teaching Hospitals NHS Foundation Trust and is coordinated by the Academic Unit of Elderly Care and Rehabilitation (Bradford Teaching Hospitals NHS Foundation Trust and University of Leeds) and the Clinical Trials Research Unit (CTRU) at the University of Leeds. The trial management group consists of the co-applicants and the teams from the coordinating units. The study will be conducted in accordance with the Research Governance Framework for Health and Social Care (2005) and CTRU standard operating procedures. The study protocol was written in line with the Standard Protocol Items: Recommendations for Interventional Trials (SPIRIT) 2013 Statement [37] and is reported in line with CONSORT 2010 statement’s extension to randomised pilot and feasibility trials [38, 39].

The SPIRIT checklist can be found in Additional file 2.

Overall trial supervision will be provided by the Trial Steering Committee (TSC) which will have an independent Chair. A sub-group of the TSC will perform a safety monitoring function since a separate Data Monitoring and Ethics Committee is not required for a feasibility trial of this nature and duration.

A patient and public involvement (PPI) group will be separately convened through existing networks at the Academic Unit of Elderly Care and Rehabilitation. They will be consulted at key time points during the trial to provide input to the content of participant materials, the acceptability of measures, any problems encountered with recruitment, interpretation of findings and dissemination of results.

Data will be entered, managed and monitored for quality and completeness by the CTRU. Missing data (except individual items collected via questionnaires) will be chased until received, confirmed as not available or the trial is at analysis. Data will be stored and managed in accordance with the provisions of the Data Protection Act (1998).

### Dissemination

Results of the study will be published in peer-review publications and will be presented at national and international conferences. We will work with the PPI group to develop lay reports to disseminate research findings to resident and relative groups and the care home staff at participating homes.

Authorship will be agreed in accordance with the PATCH trial publication policy and in line with International Committee of Medical Journal Editors recommendations.

## Discussion

The project team have built on their research experience in care homes [40, 41] to optimise implementation of this feasibility trial. This includes care home identification using the CQC database and personal engagement with a network of homes who have previously participated in research. Data collection methods focus on provision of data from multiple sources (residents, staff and routine data) to facilitate involvement of a generalisable sample of residents, i.e. those with and without capacity to consent who may or may not be able to provide information about themselves.

While this feasibility trial aims to refine recruitment of, and data collection from, care home residents and staff, a key focus relates to intervention delivery, receipt and enactment. This is a novel intervention which has not had widespread use; thus monitoring of implementation and exploration of barriers and facilitators that may hinder delivery and impact resident and staff experiences is critical. This will allow the team to further develop implementation procedures to optimise uptake in a definitive trial. Outcomes from the feasibility trial will inform processes for delivery of the definitive trial, as well as adding to the body of evidence for good practice in care home research.

### Trial status

The study is currently working to protocol version 5.0, dated 10 November 2017. Recruitment of care homes commenced in February 2017 and recruitment of residents in May 2017. As of 23 February 2018, nine care homes have been randomised with 124 residents recruited at these homes. The study has completed site selection and is projected to complete recruitment and randomisation by the end of February 2018.

## Additional files

Additional file 1: PATCH Study Progression Criteria. This document includes the red, amber and green criteria for progression to a definitive RCT. (DOCX 22 kb)

Additional file 2: PATCH SPIRIT Checklist. This is the completed SPIRIT checklist, indicating where all required elements of an interventional trial protocol are included in this manuscript. (DOC 123 kb)

Additional file 3: Information and consent materials. This document includes the most recent, REC-approved participant information and consent materials for residents, personal consultees, nominated consultees and care home staff. (PDF 1591 kb)

## Abbreviations

6CIT: Six-item Cognitive Impairment Test
ADL: Activities of Daily Living
CCAM: Continuing Care Ability Measure
CI: Confidence Interval
CQC: Care Quality Commission
CSP: Chartered Society of Physiotherapy
CTRU: Clinical Trials Research Unit
EQ-5D-5 L: EuroQol 5-Dimension 5-Level
FAC: Functional Ambulatory Classification
GP: General Practitioner
ICC: Intra-class Correlation Coefficient
MCA: Mental Capacity Act
NHS: National Health Service
PAM-RC: Physical Activity and Mobility in Residential Care scale
PATCH: Posture and mobility training in care homes
P-CAT: Person-Centred Care Assessment Tool
PPI: Patient and public involvement
RCT: Randomised controlled trial
REC: Research Ethics Committee
SCTP: Skilful Care Training Package
SD: Standard deviation
SPIRIT: Standard Protocol Items: Recommendations for Interventional Trials
TSC: Trial Steering Committee
UC: Usual care
UK: United Kingdom
