# Supplementary material for: PATCH: posture and mobility training for care staff versus usual care in care homes: study protocol for a randomised controlled trial
Source: Trials. 2018 Sep 24;19:521. doi: 10.1186/s13063-018-2863-5 (PMC6154918; doi:10.1186/s13063-018-2863-5)
Supplement: Supplementary file 1 — PATCH Study Progression Criteria. This document includes the red, amber and green criteria for progression to a definitive RCT. (DOCX 22 kb) [file 13063_2018_2863_MOESM1_ESM.docx]

**PATCH Study Progression Criteria**

**Green**

*Recruitment*

- 10 care homes are recruited; and
- On average each care home recruits at least 12 residents to the trial.

*Intervention delivery*

- At least 65% of direct care staff have attended 3 sessions; and
- At least 75% of direct care staff have attended ≥ 1 session.

*Data collection & follow-up*

- Loss to follow-up (including deaths) at 6 months is no greater than 25%;
- At least 75% of residents have outcome measures for PAM-RC and EQ-5D-5L proxy at 6-month follow-up;
- There are no safety concerns around intervention delivery or trial processes in the view of the Trial Steering Committee (TSC).

**Amber**

*Recruitment*

- 8-9 care homes are recruited; and
- On average each care home recruits 8-11 residents to the trial.

*Intervention delivery*

- At least 50% of direct care staff have attended 3 sessions; and
- At least 60% of direct care staff have attended ≥ 1 session.

*Data collection & follow-up*

- Loss to follow-up (including deaths) at 6 months is no greater than 35%;
- At least 65% of residents have outcome measures for PAM-RC and EQ-5D-5L proxy at 6 month follow-up;
- There are no major safety concerns around intervention delivery or trial processes in the view of the TSC.

**Red**

*Recruitment*

- Less than 8 care homes are recruited.

*Intervention delivery*

- Less than 50% of direct care staff have attended 3 sessions; or
- Less than 60% of direct care staff have attended ≥ 1 session.

*Data collection & follow-up*

- Loss to follow-up (including deaths) at 6 months is greater than 35%; or
- Less than 65% of residents have outcome measures for PAM-RC and EQ-5D-5L proxy at 6-month follow-up;
- There are major safety concerns around intervention delivery or trial processes in the view of the TSC.
