# Supplementary material for: PATCH: posture and mobility training for care staff versus usual care in care homes: study protocol for a randomised controlled trial
Source: Trials. 2018 Sep 24;19:521. doi: 10.1186/s13063-018-2863-5 (PMC6154918; doi:10.1186/s13063-018-2863-5)
Supplement: Supplementary file 3 — Information and consent materials. This document includes the most recent, REC-approved participant information and consent materials for residents, personal consultees, nominated consultees and care home staff. (PDF 1591 kb) [file 13063_2018_2863_MOESM3_ESM.pdf]

The Posture and Mobility in Care Homes Study

The PATCH Study

Resident Information Sheet

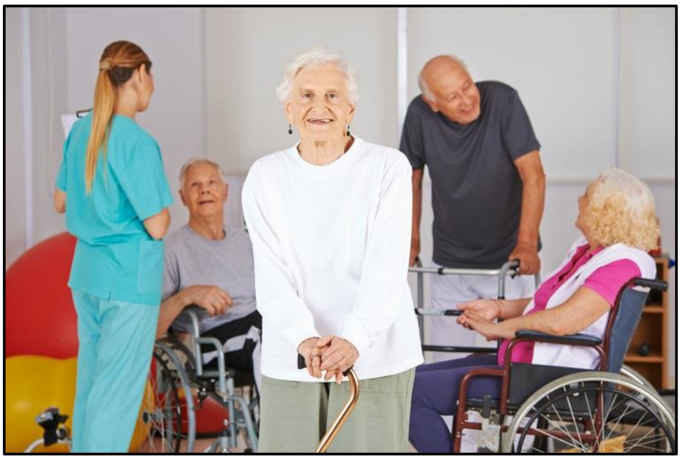

Invitation to take part in a research project

- We are inviting you to take part in a research project (also called a 'study').
- Your care home has agreed to researchers discussing the study with people who might be interested in taking part.
- Before you decide whether or not to take part, we want you to understand what this involves.
- Please read this information leaflet carefully and take time to decide whether you would like to take part. Please ask if anything is unclear.
- You are free to decide whether or not to take part. If you choose not to, it will not affect your care in any way.
- You can keep this information sheet to remind you about the study, and ask a member of staff or a researcher about the study any time you have questions.

How to contact us:

If you have any questions about this study, please contact:

<<insert name of Researchers>>
Or please contact the lead for this study:
Anne Forster: *<<insert contact details>>*

Academic Unit of Elderly Care and Rehabilitation; Bradford Institute for Health Research, Bradford Royal Infirmary, Duckworth Lane, Bradford, BD9 6RJ

Or speak to any member of staff

This project is funded by the Chartered Society of Physiotherapy

1. Why are we doing this study?

Residents in care homes may not be very active and need the help of care home staff to move about. Having good posture (e.g. sitting in a comfortable position) and keeping mobile has important health benefits. A group of physiotherapists have developed a training programme which aims to teach care home staff how to help improve residents' posture and mobility. We are doing a study where some care homes will receive the training programme and others will continue as they are now.

The study is being carried out by researchers from Bradford Teaching Hospitals NHS Trust, the University of Leeds and local physiotherapists (from Leeds Neurophysiotherapy).

2. What will happen in the study?

We are inviting 10 care homes in Yorkshire to take part. All care homes will continue to care for residents as they would normally, but some will also be provided with the new training programme. This means that the physiotherapists will visit the care home to train staff.

Separately to the physiotherapists, Study Researchers will also visit each care home. This will happen three times, and during each visit they will find out how residents and members of staff are getting on. Each visit will take place over a few weeks.

The Researchers will introduce themselves when they arrive and let you know what they are doing.

3. Why am I being asked to take part?

Your care home is involved in this study and may have the staff training. We are also inviting all residents of this care home to take part.

4. What will I be asked to do if I take part?

When the Researcher visits your care home s/he will ask if you would be willing to answer some questions. If you are happy to do this, it will take around 30 minutes on 3 or 4 occasions. S/he will also be collecting information from other residents and staff during his / her visit.

This project is funded by the Chartered Society of Physiotherapy

The Researcher will mainly ask you some questions about your activity levels (e.g. if you are able to walk around the home) and about how you have been feeling. They will also look at your posture and complete a form to show the position you are sitting or lying in, as this is an important part of the study.

Separately you may also be asked if you would be willing to talk to a researcher in more detail about your experiences of living in the care home. This would take about 20-40 minutes and, if you prefer, can happen over more than one visit. We would like to record your discussion to be sure we remember what we talked about. The researcher will only arrange (or continue) a discussion if you are happy to speak to him/her.

5. What else will happen if I take part?

The Researcher will ask a member of staff who knows you well about how you have been getting on recently. S/he will look at your care records to get some information - for example, to see if you are taking any medicines or if you have seen the doctor recently.

The Researchers may also collect information from the hospital or your GP if you have any treatment. They will make a note of your NHS number to help them get this information. The researcher will let your GP know that you are taking part in this study.

If you are happy to take part please fill out and sign the consent form we have given you. The Researcher can help you with this and answer any questions you may have. You don't need to decide now and you can discuss it with family or friends if you wish.

6. What will happen to the results of the study?

A summary of what we find out from this study will be sent to your care home and we will ask them to share this with all residents who took part in the study. The results would never include any information which would identify you or anyone else in the study in any way. We may

This project is funded by the Chartered Society of Physiotherapy

include some quotes from your conversation with the researcher, but we wouldn’t link these to your name.

We will also share the results with as many people as possible through writing articles and reports and presenting at conferences and events.

7. Will my information be safe?

Yes. We will not record your name or any personal details on any of the written information we collect or on any reports we write. We will keep any personal details about you, such as your name and date of birth, in a safe place, separately from other information we collect. Only some members of the research team will be able to look at this information. Information you and care home staff tell us about you (including information from your care home records) will be sent securely to the Clinical Trials Research Unit at the University of Leeds and the Bradford Institute of Health Research at Bradford Royal Infirmary - where it will be stored securely. Your signed consent form would be stored at the Clinical Trials Research Unit at the University of Leeds.
It is possible that the information you provide for this study may be shared with other research teams to answer new research questions in the future. If this happened your information would be entirely anonymous.

8. What if I don’t want to take part?

You don’t have to take part if you don’t want to and you don’t have to give us a reason. It will not affect the care you receive in any way.

If you are happy to take part now, but later decide to stop taking part, you can let us know by contacting us or asking your care home to do so. We will stop collecting information about you.

Whatever you decide, this will not affect the care you receive.

9. What are the advantages and risks of taking part?

This research aims to add to the understanding of what improves care for residents in care homes.

This project is funded by the Chartered Society of Physiotherapy

We hope that this study will help us to develop ways of improving posture and increasing movement of residents in care homes in the future, but we cannot say that you will definitely see a difference yourself. The same would be true if you were not part of the study.

We do not expect that there will be any risks in taking part.

10. What if there is a problem?

A group of experts in people's health and care has approved this research (number 16/YH/0114), and the care provided and research will be covered by normal insurance policies.

If you have any worries about this project you should speak to the Researcher (details on page 1), the lead for this research in this home, or any member of staff. If you remain concerned you can contact the PALS service (Patient Advise and Liaison Service, Bradford Royal Infirmary, Tel: 01274 364021)

There is no known risk in taking part in this study, but in the very unlikely case that you are harmed by taking part in this research project, there are no special compensation arrangements. If you decide to take legal action you may have to pay for it. Any claims will be subject to UK law and must be brought in the UK.

If the Researchers see or are told about any practice they feel is abusive or neglectful then this will be reported and investigated to see if any further action needs to be taken.

If it becomes apparent during discussions between you and the researcher that you are at risk of harm, or have experienced professional malpractice, the researcher will follow the local Safeguarding Adults Procedure. The researcher will talk to you about your views and wishes, including whether you wish any action to be taken. There may be circumstances when the researcher is obliged to act and breach confidentiality, including: if you lack capacity and it is in your best interests; if there is a life threatening situation; if you are subject to coercion; or if there is risk to others.

This project is funded by the Chartered Society of Physiotherapy

11. Questions

If you have any questions or would like more information, please speak to a member of staff, who will contact the Researcher.

If you would like further information about research in general, you can find more details on the following website http://enrich.nihr.ac.uk/page/understanding-research-1. If you would like a paper copy of this information to read, please ask the study researcher who will print a copy for you.

Thank you for taking the time to read this information sheet.

This project is funded by the Chartered Society of Physiotherapy

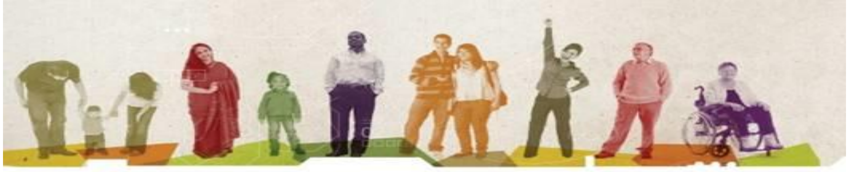

BRADFORD INSTITUTE FOR HEALTH RESEARCH | MAKING RESEARCH REAL

The Posture and Mobility in Care Homes Study

Resident Consent Form

Please read the statements below and sign at the bottom if you agree to take part.

To be completed by Researcher

Resident Trial No: _ _ _ _ _ _ Initials: _ _ _ _ DOB: _ _/_ _/_ _ _ _

Name of Care Home:…………………………………………………………………….

1.	I confirm that I have read the **Resident information sheet** dated 16th June 2017 (Version 4) for the Posture and Mobility in Care Homes study and have had the opportunity to ask questions.
2.	I understand that taking part in this study is my choice and that I am free to withdraw at any time without my care being affected.
3.	I understand that even if I withdraw from the study, the data collected from me up to that point will be used in analysing the results of the study.
4.	I understand that details of my posture, mobility and quality of life will be collected for this study. I agree to allow any information or results arising from this study to be used for further research upon the understanding that my identity will remain anonymous.
5.	I understand that data collected during the course of the study may be looked at by responsible individuals from the NHS and regulatory bodies. I understand that information held and maintained by NHS bodies may be used to provide information about my health status.
6.	I understand that my name, date of birth and NHS number will be recorded. I understand that data protection regulations will be observed and strict confidentiality maintained.
7.	I understand that a researcher might ask to talk to me about my experiences of living in the care home. If I am happy for this to go ahead, I agree to it being recorded by the researcher, and to anonymous quotes being used in research reports.
8.	I agree for my personal details and a copy of this consent form (which will include my name and date of birth) to be stored by the research team at the Clinical Trials Research Unit at the University of Leeds, and at this care home for the purposes of this study and for safe keeping.
9.	I agree to my GP being informed of my participation in this study.
10.	I agree to take part in the study.

Participant

Signature ……………………………………………………………………………………………..

Name (block capitals) ………………………………………………………………………..

Date …………………………………..

Witness (if needed)

Signature ……………………………………………………………………………………………

Name (block capitals) ………………………………………………………………………

Date ………………………………….

Researcher

I have explained the study to the above named participant and he/she has indicated his/her willingness to participate.

Signature …………………………………………………………………………………………

Name (block capitals) ……………………………………………………………………..

Date ……………………………….

(Original to be sent to the Research Office; 1 copy for care home; 1 copy for participant)

The Posture and Mobility in Care Homes Study (the PATCH study)

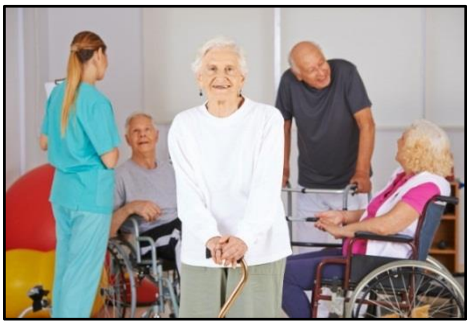

<u>Information for Personal Consultees</u>

Asking for your opinion

We would like to invite your relative / friend to be part of a research study. We feel that he / she may not be able to decide for himself / herself whether to participate in this research. We would therefore like to ask your opinion as to whether or not you think he / she would want to be involved. This is known as being a 'consultee'. We'd ask you to consider what you know of his/her wishes and feelings, and to consider his / her interests. Please let us know of any advance decisions he or she may have made about participating in research. These should take priority.

If you decide your relative/friend would have no objection to taking part, we will ask you to read and sign the consultee declaration form (your agreement to them taking part). We will then give you a copy to keep. We will keep you fully informed during the study so you can let us know if you have any concerns or you think your relative/friend no longer wishes to take part.

If you decide that your relative / friend would not wish to take part it will not affect the standard of care they receive in any way.

If you are unsure about taking the role of consultee you may seek independent advice. We will understand if you do not want to take on this responsibility.

The following information is the same as would have been provided to your relative/friend. There is a longer information sheet for residents which may be helpful too.

What is the purpose of the PATCH Study?

A group of researchers who work in Leeds and Bradford want to find out how well a new training programme for care home staff works. The training aims to help staff improve posture and mobility for residents. To find out how well it works 10 care homes will be involved in the study - half of the homes will have the staff training and half of them will continue as usual. This allows the researchers to see if there are any differences between these two groups of homes.

This project is funded by the Chartered Society of Physiotherapy

We would like to find out if the training makes a difference to residents and staff. To be able to work this out, researchers need to collect information from residents and staff, but they would only do this if residents (or their consultees) agreed to this.

What would happen if my relative / friend was part of the study?

We understand that some residents may and some may not be able to answer questions - we would like everyone to be part of the research so we get a good picture of residents' posture, mobility and quality of life.

If your relative/friend was able to speak to a researcher, she would visit the care home and arrange a good time to sit down and ask your relative / friend some questions about how he/she had been feeling. If your relative / friend was unable to do this, we would instead ask care home staff for their opinion about how he / she had been feeling over the past few days (e.g. if he / she had had any pain).

We would also like to look at residents' posture, as this is an important part of the study. The researcher would fill in a form to show your relative / friend's sitting (or lying) position.

If willing and able, your relative or friend may be asked if he/she would like to talk to another researcher in more detail about his/her life in the care home. The researcher might record this conversation so she didn't forget what they talked about. The researcher would only talk to your relative / friend if he/she didn't object and was able to do so.

If at any time your relative / friend doesn't want to do any parts of the research, he/she can just tell the researcher. The researcher will also be careful to pick up on any signs that he/she doesn't want to answer questions. He/she don't have to do anything he/she doesn't want to or isn't able to do. You can also tell us if you think he/she no longer wants to be involved.

Researchers would also ask care staff about how your relative / friend has been doing, and would collect some information from his/her care records, as well as records at the hospital or doctors' surgery. They will make a note of your relative / friend's NHS number to help them get this information. The researcher will let your relative / friend's GP know that he/she is taking part in this study.

Any information the researcher collects would be sent securely to the research team's offices for processing. The research team offices are based at the Clinical Trials Research Unit at the University of Leeds, and Bradford Institute for Health Research at Bradford Teaching Hospitals NHS Foundation Trust. If you agree to your relative / friend taking part, a copy of the form you sign will be sent to the research team's offices at the University of Leeds for safe-keeping.

The research team running this study have worked on other studies in care homes, so have a lot of experience speaking to residents and staff about research. We have found that residents often enjoy being part of research as they have one-to-one time talking to a researcher about how they are feeling.

How often would the researcher see my relative / friend?

The researcher will visit the care home at the start of the study, and then again 3 months and 6 months from now. The researcher will visit each day over a few weeks each time - to be sure that there is time to talk to all the residents and staff who are taking part in the study.

What happens at the end of the study?

All the information collected from residents and staff at all of the care homes taking part will be analysed by the research team. We will then be able to tell you what we found (the study results). These results won't identify anyone in any way. We may include some quotes from your relative / friend's conversation with the researcher, but we wouldn't link these to their name or care home (they would be anonymous).

Questions

If you have any questions, please ask a member of staff or the study researcher.

<<Insert contact details for researcher>>

This project is funded by the Chartered Society of Physiotherapy

BRADFORD INSTITUTE FOR HEALTH RESEARCH | MAKING RESEARCH REAL

The Posture and Mobility in Care Homes Study

Personal Consultee Declaration Form

Completed in relation to the wishes of: ……………………………………………………… (resident)

To be completed by Researcher
Resident Study No: _ _ _ _ _ _   Initials: _ _ _ _   DOB: _ _/_ _/_ _ _ _
Name of Care Home:…………………………………………………………………………

1.	I confirm that I have read the **Information for Personal Consultees** dated 17th July 2017 (version 4.1) for the Posture and Mobility in Care Homes study and have had the chance to ask questions. In my opinion they would have no objection to participating.
2.	I understand that my relative's/friend's (the resident) participation in this study is voluntary and that he/she is free to withdraw at any time without his/her care being affected.
3.	I understand that even if my relative/friend (the resident) withdraws from the study, the data collected from him/her up to that point will be used in analysing the results of the study.
4.	I understand that relevant sections of my relative's/friend's medical/care records and information collected during the course of the study may be looked at by members of the research team, responsible individuals from the NHS or regulatory authorities.
5.	I understand that the information held by NHS bodies (e.g. hospital or GP) may be used to provide information about my relative's/friend's health status.
6.	I understand that my relative's/friend's name, date of birth and NHS number will be recorded and details of his/her posture, mobility and quality of life will be collected for this study. I understand that any information or results arising from this study may be used for further research upon the condition that it will remain anonymous.
7.	If my relative / friend agrees to talk to a researcher about his/her experiences of living in the care home, I agree to this being recorded for use by the research team only, and to anonymous quotes being used in research reports.
8.	I agree for my relative's/friend's personal details and a copy of this declaration form (including his/her name and date of birth) to be stored by the research team at the Clinical Trials Research Unit at the University of Leeds, and the care home for the purposes of this study and for safe-keeping.
9.	I agree to my relative/friend's GP being informed of their participation in this study.
10.	I agree for my name and contact information to be held by the research team at the Clinical Trials Research Unit at the University of Leeds for the purposes of this study and for safe-keeping.

OPTION 1 – Agreeing to participation
In my opinion, my relative/friend would want to take part in this study:

Please tick this box ☐

Please sign and date this form and return it to the researcher.

OPTION 2 – Not agreeing to participation

If you feel your relative/friend **would not** have wanted to take part in this study,

please tick this box ☐

Please sign and date this form and return it to the researcher

Note: if we don't hear back from you, we will talk to a member of staff within the care home to make an assessment of your relative/friend's views on participation in this study

OPTION 3 - Uncertain

If you feel you are **unable to make a judgment** on your relative/friend's interest in taking part in this study please tick this box ☐

Please sign and date this form and return it to the researcher.

Please sign and return this form in the pre-paid envelope provided. Thank you.

Your details:

Your Signature ……………………………………………………………………………………………………

Your Name (block capitals) ……………………………………………………………………………………

Your address ……………………………………………………………………………………………………….

Your Telephone Number………………………………………………………….

Today's Date ……………………………………………

Your relationship to the resident ……………………………………………………………………….

Researcher
I confirm the above named consultee received all relevant information sheets and declaration form by post and was given the opportunity to discuss the study.
Signature ………
Name (block capitals) ……………………………………………………………………………………………
Date ……………………………………………………

(Original to be sent to the Research Office; 1 copy for care home, 1 copy for consultee)

The Posture and Mobility in Care Homes Study (the PATCH study)

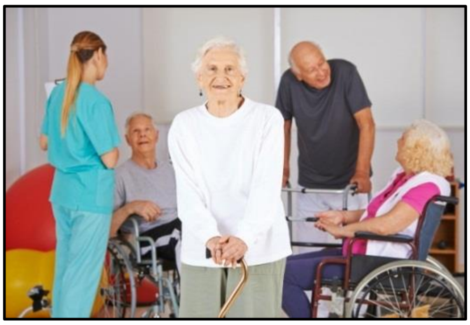

Information for Nominated Consultees

Asking for your opinion

We would like to invite one or more residents in your care home to take part in a research study. We feel that they may not be able to decide for themselves whether to participate in this research. We would therefore like to ask your opinion as to whether or not you think they would want to be involved. This is known as being a 'consultee'. We'd ask you to consider what you know of the residents' wishes and feelings, and to consider their interests. Please let us know of any advance decisions they may have made about participating in research. These should take priority.

In deciding what you think a resident's wishes would be if they had capacity, you should attempt to seek the views of the person, and also the views of their family or friends who may be unwilling or unable to act as a consultee.

After appropriate consultation we would like you to complete the consultee declaration form. You can indicate on this form whether you feel the named person would or would not have wished to participate. We will then give you a copy to keep. If the resident does take part in the research we will keep you fully informed during the study so you can let us know if you have any concerns or you think the resident no longer wishes to take part.

If you are unsure about taking the role of consultee you may seek independent advice. We will understand if you do not want to take on this responsibility, and this will not affect your employment or the resident's future involvement in the study. We will work with the care home manager to identify another staff member to take on this role.

The following information is the same as would have been provided to a resident. There is also a longer information sheet for residents which may help you decide.

What is the purpose of the PATCH Study?

A group of researchers who work in Leeds and Bradford want to find out how well a new training programme for care home staff works. The training aims to help staff improve posture and mobility for residents. To find out how well it works 10 care homes will be involved in the study - half of the homes will have the staff training and half of them will continue as usual. This allows the researchers to see if there are any differences between these two groups of homes.

This project is funded by the Chartered Society of Physiotherapy

We would like to find out if the training makes a difference to residents and staff. To be able to work this out, researchers need to collect information from residents and staff, but they would only do this if residents agreed to this.

What happens if a resident takes part in the study?

A researcher would visit the care home, and arrange a good time to sit down and ask the resident some questions about how he/she had been feeling. We would also like to look at residents' posture, as this is an important part of the study. The researcher would fill in a form to show the resident's sitting (or lying) position.

The resident may be asked if he/she would like to talk to another researcher in more detail about his/her life in the care home. The researcher might record this conversation so she didn't forget what they talked about. The researcher would only talk to the resident if he/she didn't object.

If at any time the resident doesn't want to do any parts of the research, they can just tell the researcher. The researcher will also be careful to pick up on any signs that they don't want to answer questions. They don't have to do anything they don't want to. You can also tell us if you think they no longer want to be involved.

Researchers would also ask care staff about how the resident has been doing, and would collect some information from his/her care records, as well as records at the hospital or doctors' surgery. They will make a note of the resident's NHS number to help them get this information. The researcher will let the resident's GP know that he/she is taking part in this study.

Any information the researcher collects would be sent securely to the research team offices for processing. The research team offices are based at the Clinical Trials Research Unit at the University of Leeds, and Bradford Institute for Health Research at Bradford Teaching Hospitals NHS Foundation Trust. If you agree to the resident taking part in this study, the form you sign will be sent to the research team's offices at the University of Leeds for safe keeping.

The research team running this study have worked on other studies in care homes, so have a lot of experience speaking to residents about research. We have found that they often enjoy being part of research as they have one-to-one time talking to a researcher about how they are feeling.

How often would the researcher see each resident?

The researcher will visit the care home at the start of the study, and then again 3 months and 6 months from now. The researcher will visit each day over a few weeks each time - to be sure that there is time to talk to all the residents and staff who are taking part in the study.

What happens at the end of the study?

All the information collected from residents and staff at all of the care homes taking part will be analysed by the research team. We will then be able to tell you what we found (the study results). These results won't identify anyone in any way. We may include some quotes from the resident's conversation with the researcher, but we wouldn't link these to their name or to the care home (they would be anonymous).

This project is funded by the Chartered Society of Physiotherapy

Questions

If you have any questions, please ask a member of staff or the study researcher.

<<Insert contact details for researcher>>

This project is funded by the Chartered Society of Physiotherapy

BRADFORD INSTITUTE FOR HEALTH RESEARCH
MAKING RESEARCH REAL

The Posture and Mobility in Care Homes Study

Nominated Consultee Declaration Form

To be completed by Researcher
Resident Study No: _ _ _ _ _ _ Initials: _ _ _ _ DOB: _ _/_ _/_ _ _ _
Name of Care Home:……………………………………………………………………

Completed in relation to the wishes of: …………………………………………………… (resident)

1.	I confirm that I have read the **Nominated Consultee information sheet** dated 16th June 2017 (Version 4.0.) for the Posture and Mobility in Care Homes study and have had the chance to ask questions. In my opinion the resident named above would have no objection to participating.
2.	I understand that the named resident's participation in this study is voluntary and that he/she is free to withdraw at any time without his/her care being affected.
3.	I understand that even if the named resident withdraws from the study, the data collected from him/her up to that point will be used in analysing the results of the study.
4.	I understand that relevant sections of the named resident's medical/care records and data collected during the course of the study may be looked at by members of the research team, responsible individuals from the NHS or regulatory authorities.
5.	I understand that the information held by NHS bodies (e.g. hospital or GP) may be used to provide information about the named resident's health status.
6.	I understand that the named resident's name, date of birth and NHS number will be recorded and details of his/her posture, mobility and quality of life will be collected for this study. I understand that any information or results arising from this study may be used for further research upon the condition that it will remain anonymous.
7.	If the named resident agrees to talk to a researcher about his/her experiences of living in the care home, I agree to this being recorded for use by the research team only, and to anonymous quotes being used in research reports.
8.	I agree for the named resident's personal details and a copy of this declaration form (including the resident's name and date of birth) to be stored by the research team at the Clinical Trials Research Unit at the University of Leeds, and this Care Home for the purposes of this study, and for safe-keeping
9.	I agree to the named resident's GP being informed of their participation in this study.
10.	I agree for my name and contact information to be held by the research team at the Clinical Trials Research Unit at the University of Leeds for the purposes of this study and for safe-keeping.

OPTION 1 - Agreeing to participation

In my opinion the named resident **would** want to take part in this study:

Please tick this box ☐

Please sign and date this form and return it to the researcher

OPTION 2 - Not agreeing to participation

If you feel the named resident **would not** have wanted to take part in this study, please tick this box ☐

Please sign and date this form and return it to the researcher

OPTION 3 - Uncertain

If you feel you are **unable to make a judgment** on the named resident's interest in taking part in this study please tick this box ☐

Please sign and date this form and return it to the researcher.

Nominated Consultee

Signature ………

Name (block capitals) ……………………………………………………………………………………

Date ……………………………………………

Trial ID …………………………….. (to be completed by the researcher)

Researcher

I have explained the study to the above named consultee and he/she has indicated that in his/her opinion that the named resident would/would not (delete as appropriate) be willing to participate.

Signature ……………………………………………………………………

Name (block capitals) …………………………………………………

Date …………………………

(Original to be sent to the Research Office; 1 copy for care home)

UNIVERSITY OF LEEDS

Leeds Neurophysiotherapy

ctru

Bradford Teaching Hospitals NHS Foundation Trust

BRADFORD INSTITUTE FOR HEALTH RESEARCH

The Posture and Mobility in Care Homes Study

Staff Information Sheet

Invitation to participate

- We are inviting you to take part in a research project.
- Before you decide whether or not you wish to participate we would like to explain why the research is being done and what it will involve.
- Please read this information leaflet carefully and take time to decide whether or not to take part.
- You are free to decide whether or not to take part. If you choose not to, this will not affect your employment in any way.
- If you decide to take part, you should keep this information sheet for future reference
- Ask us if anything is unclear, or if you would like more information.

(Please turn over)

Contents

1. Why are we doing this study?
2. Who is carrying out the study?
3. What will happen in the study and what will I need to do?
4. Who will see the information I provide?
5. What if I don't want to take part?
6. What are the advantages and risks of taking part?
7. More information about taking part
8. Questions

How to contact us:

If you have any questions about this study, please contact:
Name of Researchers:
<<insert>>
Or please contact the lead for this study:
Anne Forster: <<insert contact details>>

This project was funded by the Chartered Society of Physiotherapy

1. Why are we doing this study?

Residents in care homes may be inactive and require the help of care home staff to move about. Maintaining good posture and keeping mobile has important health benefits. A group of physiotherapists have developed a training programme which aims to teach care home staff how to improve the posture of residents and enable mobility. We are conducting a study where some care homes will receive the training programme and others will continue as they are now.

2. Who is carrying out the study?

The study is being carried out by researchers at Bradford Teaching Hospitals NHS Foundation Trust, the University of Leeds and local physiotherapists.

3. What will happen in the study and what will I need to do?

*(The **black** writing describes what will happen and the **blue** writing highlights the parts we would like you and / or other members of staff to help with.)*

Involving care homes in Yorkshire

We plan to ask 10 care homes across Yorkshire to take part in this research. Five of the care homes will carry on delivering care as usual, while five will be randomly chosen to be receive the new training programme.

We will collect information from all 10 care homes so we can find out how residents and staff found the new approach – and so we can compare those homes that use the new training programme with those that don't.

Resident consent

Before it is decided whether or not a care home will use the new training programme, residents will be asked to provide their consent to be involved in this study. We may also ask a relative or staff member to act as a 'consultee' to provide agreement on a resident's behalf if they are unable to make the decision themselves. If they give consent (or there is agreement) to be involved, this will mean a researcher from the study will visit to collect some information about their mobility and quality of life, and ask them some questions on three occasions over 6 months. We will not involve anyone in the study who has not agreed to this and they can change their mind about taking part at any time.

Involving staff as 'consultees'

This project was funded by the Chartered Society of Physiotherapy

Where a resident isn't able to make a decision for themselves about taking part in the study (e.g. if they have problems with memory or understanding), we will try to contact a relative or friend who could help them make that decision. (A relative or friend would be known as a 'personal consultee'). Where a relative or friend isn't available to help in this way, we may ask you to be a consultee for a resident. By doing this you would be thinking about whether you feel they would want to take part if they *were* able to make the decision themselves. If you are asked to do this, we would give you a separate information sheet which explains in more detail what it means to be a consultee. You wouldn't have to do this if you didn't want to.

Care Home 'Allocation' - Usual Care or posture and mobility training

Once all residents have had the opportunity to join the study, care homes will be allocated to receive a posture and mobility training programme (called Skilful Care) or to continue with 'usual care' (i.e. carrying on doing what you would normally do). The best way to compare the two approaches is to decide by chance ('randomly') who gets which one. So in this study your care home will be either part of the 'usual care' group of homes or the group of homes being provided with the Skilful care training. At the end of the study these two groups can be compared to see whether one approach is more helpful than the other.

The information we collect from both the 'Skilful Care' and 'usual care' homes is equally important for the research.

Posture and mobility training (Skilful Care, the intervention)

A group of senior physiotherapists have developed a training package for care home staff called the Skilful Care Training Package (SCTP).

The training course aims to increase the skills of care assistants and nursing staff in a) handling (ways to increase movement) and b) good positioning (in sitting or lying). The aim is to protect body shape, reduce the risk of choking, reduce pain and enable residents to be as active and independent as they are able to be. The training is generally presented in three 2½ hour sessions, and would take place within the care home. If your care home is offered the Skilful Care Training Package we would like all care and nursing staff to attend. Your manager will make sure you have time to go to the training sessions within your working hours. We will collect some information on how many people attend to check that people do have time to go. You will also be asked to try out the new skills you learn in your everyday care work with residents.

Researcher visits

All research projects need information (data) to be able to report results at the end of the study. For this study a researcher will visit your care home to collect information from residents about how they are doing, as well as to collect some information from staff members and from the care home records.

This project was funded by the Chartered Society of Physiotherapy

The study researcher will visit each care home to collect information three times over 6 months (at the start, 3 months later and 6 months later). One researcher will visit the care home for around two to four weeks to collect this information at each time point.

As a number of the residents living in this home will be taking part in this study we need to gather information about how they are doing at each researcher visit.

We will ask residents to fill in some questionnaires with the researcher at each visit. These ask about their quality of life, movement and how they have been feeling. We will talk to the resident where possible and ask them how they are doing.

We will also ask some staff to help by providing information about residents' mobility and quality of life.

If you are happy to provide information about a resident(s) you know well, the researcher will sit with you and ask you questions about the resident or ask you to complete a questionnaire. This should take no more than 30 minutes for each resident. We can do this at a time convenient to you during the period of time that the researcher visits. The home's management team has agreed staff can fill out these forms with a researcher during normal working hours.

We will ask all care and nursing staff (if happy to do so) to complete a questionnaire about themselves which will take about 10 minutes.

We will assume that you providing data (about a resident and / or yourself) indicates your willingness for this information to be used for the research project, as detailed in this information sheet.

If your care home has the training programme, the researcher may undertake interviews with some staff, relatives and residents to find out what aspects of the training worked well and which aspects could be improved. If you are invited to be involved in this, more information will be provided and you will be asked to sign a consent form.

4. Who will see the information I provide?

Only the research team will see any information collected about you or the resident and it will not be shared with anyone you work with, the resident or their family.

Completion of all questionnaires is confidential, which means we won't record your name alongside the answers you give and care home managers/colleagues will not see it.

This project was funded by the Chartered Society of Physiotherapy

If you are asked to provide any data relating to a resident(s) you know well, and you are happy to do this, we will ask for some basic information (e.g. your name and job role) so we can speak to you again at future data collection points.

5. What if I don't want to take part?

We would like all nursing and care staff to participate in the training if it is offered in your home. You are free to decide whether or not you wish to provide data about yourself or a resident you know well. If you decide not to take part you do not have to give a reason and this will not affect your employment in any way.

Staff questionnaires will be circulated to all nursing and care staff at each time point; however, if you do not wish to provide this data, do not return the questionnaire.

6. What are the advantages and disadvantages of taking part?

Taking part will help us gain an understanding of how the training might be helpful for staff and residents in care homes and so may help residents in the future.

We do not expect there will be any risks in taking part.

7. More information about taking part

What will happen if I do not want to carry on providing data?

You can choose not to provide data about yourself at any time point by simply not returning the staff questionnaire. You can also decide against providing data relating to a resident at any time point - this will not affect your employment in any way. In these instances the researcher will ask the management team to identify another staff member who knows the resident well. Any information already collected from you for the study will remain on file and will be included in the final study analysis. At the end of the study, data we collect from you and from residents will be securely stored for a minimum of 5 years. Arrangements for confidential destruction will then be made.

Will my taking part be kept confidential?

If you decide to provide data for the study, the written information collected about you, the care home, and the residents during the course of the study will be kept strictly confidential. The information collected will be recorded on paper forms and sent securely from your care home to the research offices at the University of Leeds and the Bradford Institute of Health Research. This information will be securely stored at the Research Office in Leeds on paper and electronically, under the provisions of the 1998 Data Protection Act. At the end of the study, your data will be securely archived for a minimum of 5 years. Arrangements for confidential destruction will then be made.

It is possible that the information you provide for this study may be shared with other research teams to answer new research questions in the future. If this happens the information would be anonymised, meaning your care home name, staff and resident names would never be included, so that no-one would be able to identify you or any study participants from it. Other research teams would not be given any personal details about who participated in the study.

If the researcher sees or is informed of any malpractice, the researcher will follow local safeguarding Adult's procedures. Under such circumstances, it may be that the researcher is obliged to act and breach confidentiality.

What will happen to the results of the study?
The results of the study will be shared with people living in care homes and their families, care provider organisations and their staff, policy makers, researchers and the general public. We will share the results through writing articles for magazines and journals, through speaking at conferences and other public events, through producing information leaflets and through the web-pages of the organisations involved. No information that might identify you will ever be included in information we share about the study results.

We will produce a specific information leaflet about the results of the study for staff. These will be sent to the care home. If you leave the care home before the study ends, you can ask for us to send you a copy to an address of your choice when the study ends.

What if there is a problem?
If you have a concern about any aspect of this study, you should ask to speak with the Researcher who will do their best to answer your questions. If you remain unhappy you may wish to contact the PALS service (Patient Advise and Liaison Service at Bradford Royal Infirmary, Tel: 01274 364021).

If you are harmed by taking part in this research project, there are no special compensation arrangements. If you decide to take legal action you may have to pay for it. Any claims will be subject to UK law and must be brought in the UK.

If the Researchers see any practice they feel is abusive or neglectful then this will be reported and investigated to see if any further action needs to be taken.

Who is organising, funding and reviewing the research?
The study is being organised and supervised by Bradford Teaching Hospitals and the University of Leeds. It is funded by research funding from the Chartered Society of Physiotherapy.

All research is looked at by an independent group of people called a Research Ethics Committee to protect the safety, rights, wellbeing and dignity of those taking part. This study has been reviewed and approved by Yorkshire & the Humber - Leeds East Research Ethics Committee (Ref: 16/YH/0114)

8. Questions?

If you have any questions or would like more information, you can speak to the Researcher, whose details can be found on page 1 of this information sheet.

If you would like further information about research in general, you can find more details on the following website: http://enrich.nihr.ac.uk/page/understanding-research-1. If you would like a paper copy of this information to read, please ask the study researcher who will print a copy for you.

Thank you for taking the time to read this information sheet.

This project was funded by the Chartered Society of Physiotherapy
